# Review of the application of dual‐energy CT combined with radiomics in the diagnosis and analysis of lung cancer

**DOI:** 10.1002/acm2.70020

**Published:** 2025-02-17

**Authors:** Rongyu Zhang, Hao Zheng, Jie Lin, Junna Wang

**Affiliations:** ^1^ Department of Radiology The First Affiliated Hospital of Zhejiang Chinese Medical University (Zhejiang Provincial Hospital of Chinese Medicine) Hangzhou China; ^2^ The First School of Clinical Medicine Zhejiang Chinese Medical University Hangzhou China

**Keywords:** deep learning, dual‐energy CT, lung cancer, radiomics

## Abstract

Lung cancer is one of the most common malignant tumors in the world. Early detection and precise treatment are of great significance to clinical decision‐making and patient prognosis. As an emerging imaging technology, dual‐energy computed tomography (DECT) has increasingly prominent advantages in multi‐parameter and quantitative analysis in assessing the benign and malignant, classification, and prognosis of lung cancer. Radiomics uses an automated high‐throughput method to extract a large number of quantitative features from medical images, quantify tumor heterogeneity, monitor tumor development and prognosis, and provide new ideas for the diagnosis and identification of lung cancer. This article will review the application progress of DECT post‐processing technology combined with radiomics in lung cancer diagnosis, identification, biomarker and gene prediction, and prognosis assessment.

## 1 INTRODUCTION

Lung cancer is one of the most common malignant tumors in the world. Non‐small cell lung cancer (NSCLC) accounts for approximately 85% of all lung cancer cases, and adenocarcinoma is the main histological type.^1^ According to Cancer statistics, 2024, the number of deaths from lung cancer every year still exceeds that of colorectal cancer, breast cancer, and prostate cancer combined.^2^ Therefore, early diagnosis and pathological classification of lung cancer are of great significance to improving patient prognosis and quality of life. Conventional computed tomography is currently the most commonly used imaging method for lung cancer, but it has limitations in distinguishing tissues with similar densities. Dual‐energy computed tomography (DECT), which has emerged in recent years, extends conventional CT functions beyond density measurement to obtain dual‐energy images with fast imaging speed, high quality, and low radiation. It provides multi‐parameter imaging, which is a major change in the history of CT development.^3^ Radiomics uses high‐throughput technology to extract quantitative features of medical images and obtain tumor information that is indistinguishable to the human eye, providing new ideas for the diagnosis and identification of lung cancer.^4^ This review explores recent developments in the integration of DECT post‐processing and radiomics, focusing on their application in diagnosing lung cancer, identifying biomarkers, predicting genetic alterations, and assessing prognosis.

**FIGURE 1:**
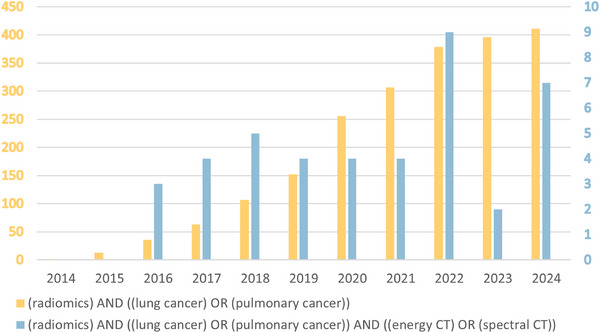
Number of publications by year from 2014 to 2024. The search was from PubMed for (radiomics) AND [(lung cancer) OR (pulmonary cancer)] and (radiomics) AND [(lung cancer) OR (pulmonary cancer)] AND [(energy CT) OR (spectral CT)]. The field of radiomics has been growing steadily over the past decade, but studies incorporating DECT are few and the growth is erratic (Accessed on December 5, 2024).

We performed a literature search on publications from 2014 to the end of 2024 from PubMed. The use of the keyword (radiomics) returned 13 120 results, adding [(lung cancer) OR (pulmonary cancer)] returned 1884 results, then adding [(energy CT) OR (spectral CT)] returned 38 results. Figure 1 illustrates the number of publications annually for the literature search. We screened these publications and excluded two PET/CT and one abdominal PCCT‐related articles, one review article, and 20 publications not directly related to the study of DECT combined with radiomics in the diagnosis and identification of lung cancer (e.g., new framework study to improve the robustness of radiomics, interobserver agreement of radiomic characteristics of subsolid pulmonary nodules), leaving a total of 14 results. In order to further explore the application of deep learning combined with DECT in the differential diagnosis of lung cancer, we conducted a literature search on publications in PubMed from 2014 to the end of 2024. Using the keyword (deep learning) returned 88 991 results, adding [(lung cancer) OR (pulmonary cancer)] returned 2317 results, and then adding [(energy CT) OR (spectral CT)] returns 21 results. Seventeen publications not directly related to DECT and differential diagnosis of lung cancer are excluded things, leaving a total of four results. In addition, we searched CNKI using the same search method, excluding master's theses, leaving a total of five results. To the best of our knowledge, there is no comprehensive review that examines feature selection methods and predictive models used in dual‐energy CT lung cancer radiomics. Hence, we provide a review regarding this topic based on a thorough analysis of existing literature. Table 1 is a summary of the above publications that meet the inclusion criteria.

**TABLE 1:** Excerpts from lung cancer radiomics research report.

Literature	Purpose	No. of patients	Energy spectrum parameters	Method	Research result
Xu et al.^19^	Predict benign and malignant pulmonary nodules	123	IC	LDA	The iodine‐based histological prediction model achieved superior diagnostic efficacy (AUC = 0.834).
Xu et al.^20^		194	IC	LR	The joint model has the highest diagnostic ability (AUC = 0.912).
Liang et al.^21^		153	VMI (70 keV)	PAC LR	The radiomics model that combines arterial phase and venous phase imaging has an AUC of 0.8772.
Xu et al.^22^		242	VMI (65 keV)	LR	AP and VP based on 65 keV radiomics score performed best in distinguishing benign and malignant SPSN (AUC 65 keV‐AP = 0.92, AUC 65 keV‐VP = 0.88).
Chang et al.^23^		113	ED	LR	The joint model established using radiomic features, ED, and vacuole sign has high specificity and sensitivity for distinguishing benign and malignant pGGNs.
Azour et al.^25^	Distinguish pathological types of lung cancer	100	IC	Benjamini‐Hochberg	Radiomic signature analysis combined with DECT can provide discriminatory capabilities beyond traditional iodine quantification analysis.
Chen et al.^4^		129	VMI (40 and 100 keV)	LR	The AUC of the joint model in the training group and validation group were 0.93 and 0.90, respectively.
Li et al.^26^		162	VMI (70 and 120 keV)	LR	The AUCs of the omics model retraining group and validation group were 0.77 and 0.74, respectively.
Zheng et al.^1^	Predicting invasiveness of lung adenocarcinoma	92	VMI (50 and 150 keV)	LR	The AUC of the omics model in predicting the invasiveness of lung adenocarcinoma reached 0.929, showing better accuracy, sensitivity, and specificity than the clinical‐DECT model.
Chang et al.^27^		176	PI VMI (60 keV)EDM	SVM LR LR‐Lasso	The AUC of the joint model constructed by LR‐Lasso reached 0.944, which is helpful for predicting the invasiveness of GGNs before surgery.
Zheng et al.^28^		107	VMI (50 and 150 keV)	LR‐LASSO	The DECT‐based radiomics model showed excellent performance in predicting tumor differentiation, with AUCs of 0.997 and 0.743 in the training set and test set, respectively.
Zheng et al.^32^	Predicting biomarkers	220	IC Zeff VMI (40 keV) EDM	LR SVM	The predictive performance of the combined model for PD‐L1≥1% reaches 0.917.
Ma et al.^35^	Predicting lung cancer mutation status	175	CT values (HU) Water Concentration IC	LR RF AdaBoost	The prediction model based on clinical, DECT, and radiomics features was the best model to predict LUAD EGFR mutation status (AUC of training and validation groups were 0.86 and 0.83, respectively).
Wu et al.^36^		73	λHU	LR	The accuracy of the model in predicting EGFR expression was higher than that in predicting VEGF expression.
Zhou et al.^37^		103	NIC λHU	LR	The AUC of the combined model in predicting EGFR gene mutations in lung adenocarcinoma reached 0.827.
Choe J et al.^41^	Evaluate the treatment effect and prognosis of lung cancer	93	IOM	Cox	The radiomics signature (histogram entropy) was an independent risk factor for predicting overall survival and disease‐free survival, and its addition to clinical stage improved prediction of overall survival (C‐index, 0.72 for OS and 0.70 for DFS).
Wu et al.^42^		98	IC ED λHU	LASSO	The clinical‐radiomics nomogram based on pre‐treatment DECT showed good performance in predicting clinical response to non‐surgical therapy in NSCLC. (AUC: 0.87 and 0.85 in training and validation sets, respectively)
Wang et al.^44^	Dual‐energy CT imaging based on deep learning	125	IC VNC ED Zeff	CNN	The AUC of the joint model was 0.932 and 0.887, respectively.
Yu et al.^45^		141	VMI (70 keV)	ANN	The AUCs in the training group and validation group were 0.888 and 0.871, respectively.
Vinay Kumar et al.^46^		36	λHU	CNN	By using an improved U‐Net deep learning model, combined with dual‐energy CT and radiomics features, the accuracy of automatic detection of lung cancer nodules in non‐enhanced chest CT images can be significantly improved.
Ma et al.^47^		130	VMI (40‐80 keV)	CNN	AI algorithms trained on CPI show consistent diagnostic performance on VMI. 80 keV may be the optimal virtual monochromatic energy for identifying preoperative IAC on non‐contrast chest CT.
Wang et al.^48^, ^49^		215	VMI (40‐140 keV)	CNN	The use of multi‐level fusion improved the accuracy of predicting lung cancer lymph node metastasis to 93%, with a Kappa value of 86%.

Abbreviations: λHU**, **slope of the spectral Hounsfield unit curve; AdaBoost, Bayes and adaptive boosting; ANN, artificial neural network; CNN, convolutional neural network; EDM, electron density map; IC, iodine concentration; IOM , iodine overlay map; LDA**, **linear discriminant analysis; LR, logistic regression; LUAD, lung adenocarcinoma; PAC, principal component analysis; pGGNs, pure ground‐glass nodules; PI, conventional 120 kVp poly energetic images; NIC, normalized iodine concentrations; RF, random forest; SVM, support vector machine; VMI,= virtual monoenergetic images; VNC, virtual non‐contrast image; Zeff, effective atomic number.

## 2 DUAL‐ENERGY CT MULTI‐PARAMETER IMAGING

Generally speaking, due to differences in elemental composition, substances have different linear attenuation coefficients at different energy levels and exhibit different CT values. However, the CT values of different substances may overlap, making it difficult to distinguish them on traditional single‐energy CT. Alvarez and Macovski^5^ defined a formula for calculating the role of the photoelectric effect and the Compton effect in the tissue attenuation coefficient (μ) for a given energy (E).^5^

(1) $$\mu \;\left(E\right)=\mu_{p}\;\left(E\right)+\mu_{c}\;\left(E\right)=\alpha_{P}\;f_{P}\left(E\right)+\alpha_{c}f_{c}\left(E\right)$$

$f_{P}\left(E\right)$ and $f_{c}\left(E\right)$ only depend on the energy of the photon beam and are two known functions. $\alpha_{P}$ and $\alpha_{c}$ are coefficients describing the contributions of the photoelectric effect and the Compton effect, respectively. These values only depend on the atomic number of the tissue. By collecting data of two different energies, the equation containing the two unknowns $\alpha_{P}$ and $\alpha_{c}$ can be solved, thereby achieving the purpose of qualitatively distinguishing substances.

(2) $$\mu \;\left(LE\right)=\alpha_{P}\;f_{P}\left(LE\right)+\alpha_{c}f_{c}\left(LE\right)$$

(3) $$\mu \;\left(HE\right)=\alpha_{P}\;f_{P}\left(HE\right)+\alpha_{c}f_{c}\left(HE\right)$$

**FIGURE 2:**
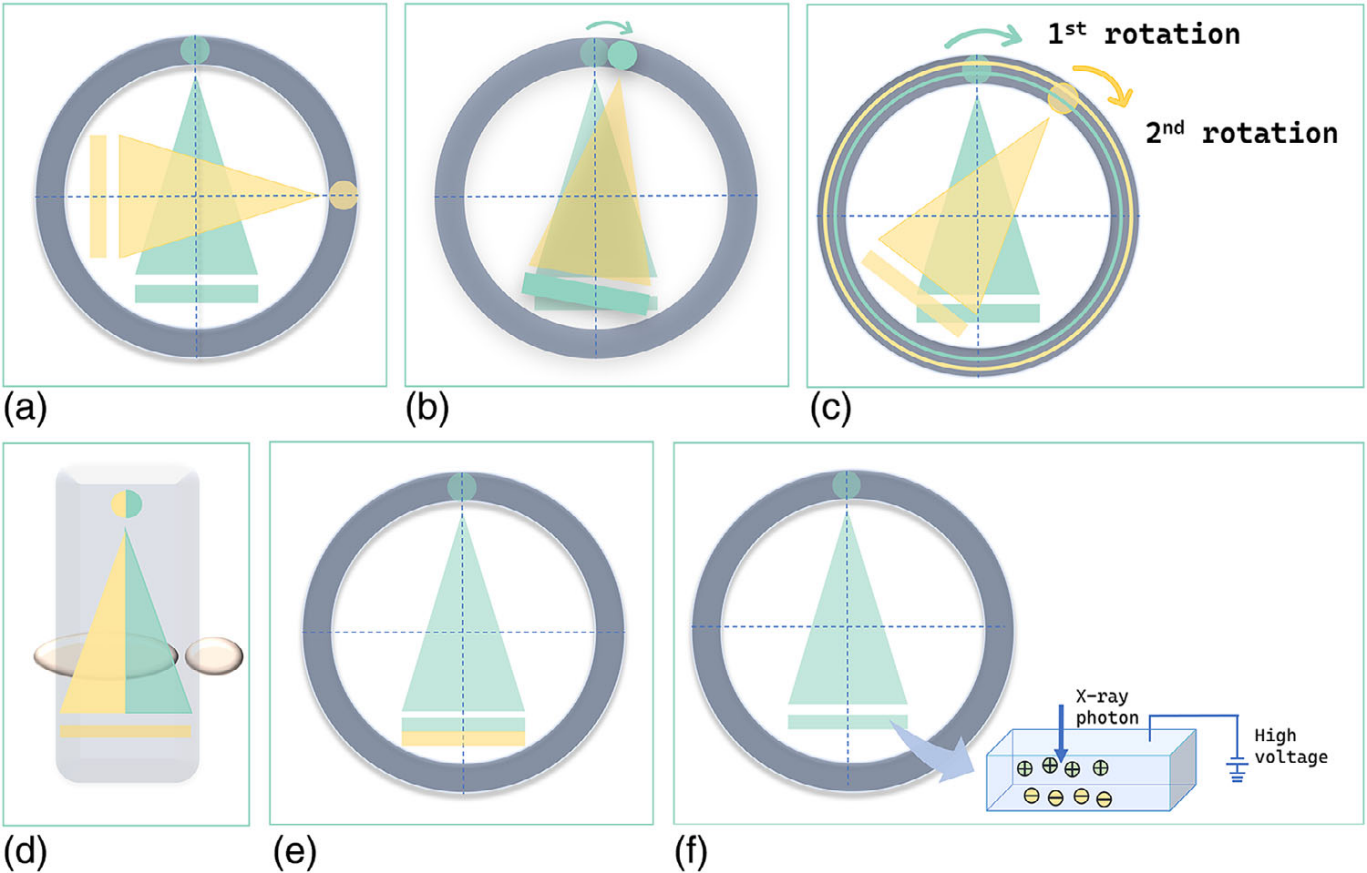
CT scanner systems that are currently available for dual‐energy imaging. (a) Dual‐source. (b) Single‐source with ultrafast kV switching. (c) Single‐source without ultrafast kV switching. (d) Single‐source with split‐filter. (e) Single‐source with dual‐layer detector. (f) Single‐source with photon‐counting detector. CT, computed tomography.

DECT is based on detecting spectra of two different energies, high and low, and can provide objective and quantitative multi‐parameter information, such as iodine overlay map (IOM), virtual non‐contrast image (VNC), effective atomic numbers (Zeff), virtual monoenergetic images (VMI), electron density diagram (ED), and energy spectrum curves. These image sets can complement and in some areas overcome the limitations posed by traditional CT. Several multi‐energy CT scanner types have been developed, which can be broadly classified as source‐based or detector‐based based on the predominant method of spectral separation. Source‐based scanners include dual source (Figure 2a), fast kilovolt switching (Figure 2b), dual spin (Figure 2c), and beam splitting technologies (Figure 2d). Detector‐based scanners include dual layer (Figure 2e) and photon counting scanners (Figure 2f)^6^.

### 2.1 IOM and VNC image

**FIGURE 3:**
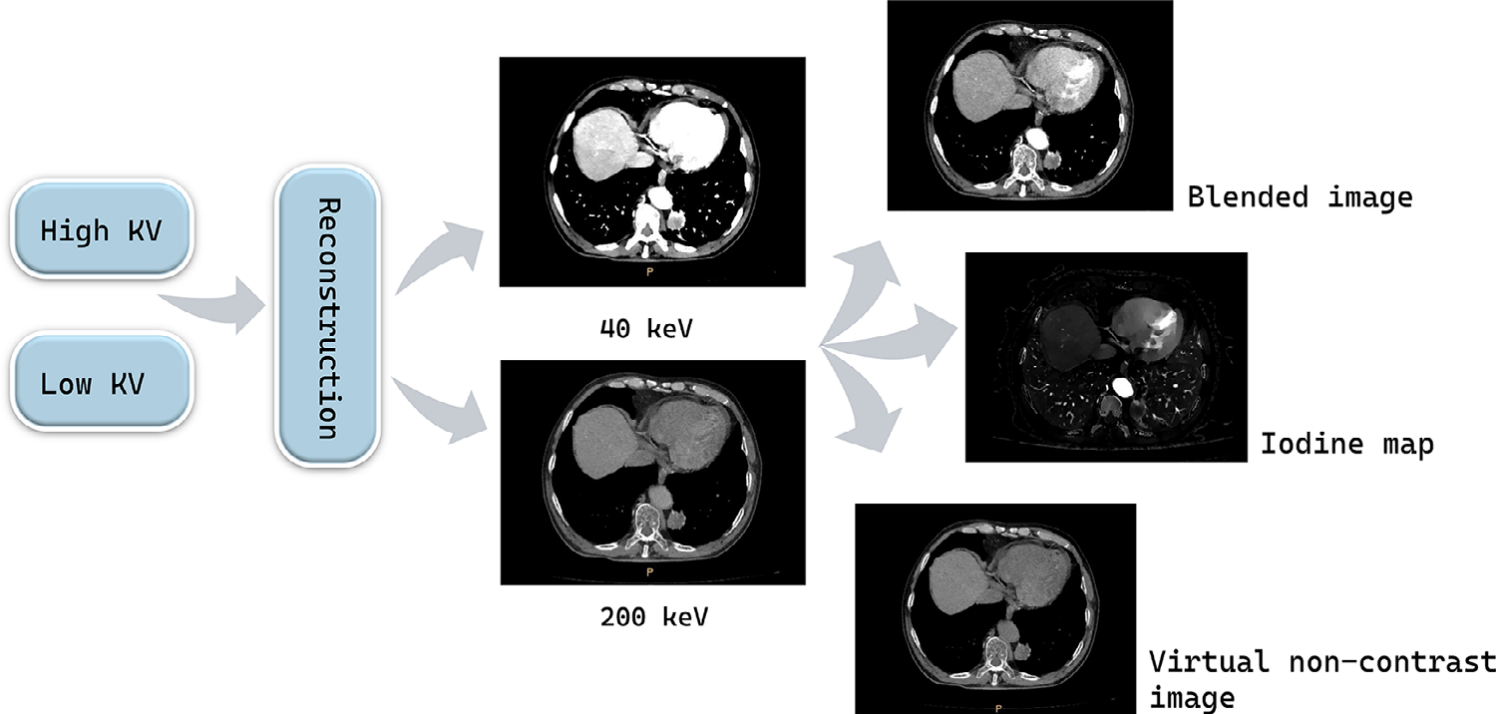
Image‐based approach for dual‐energy CT analysis.

Dual‐energy analysis methods can be divided into analysis based on image data and analysis based on raw data.^7^ Analysis based on image data obtains weighted average images at various tube voltages by mixing high‐energy images and low‐energy images, creates iodine mapping images through material separation, and removes iodine components in CT contrast‐enhanced images to obtain VNC^8^ (Figure 3). IOM have shown high practicability in distinguishing benign and malignant lung tumors by accurately displaying lesion volume and blood perfusion information.^9^ The latest research shows that VNC images have advantages in identifying lung hamartomas and malignant tumors and avoid additional radiation exposure.^10^

### 2.2 VMI

**FIGURE 4:**
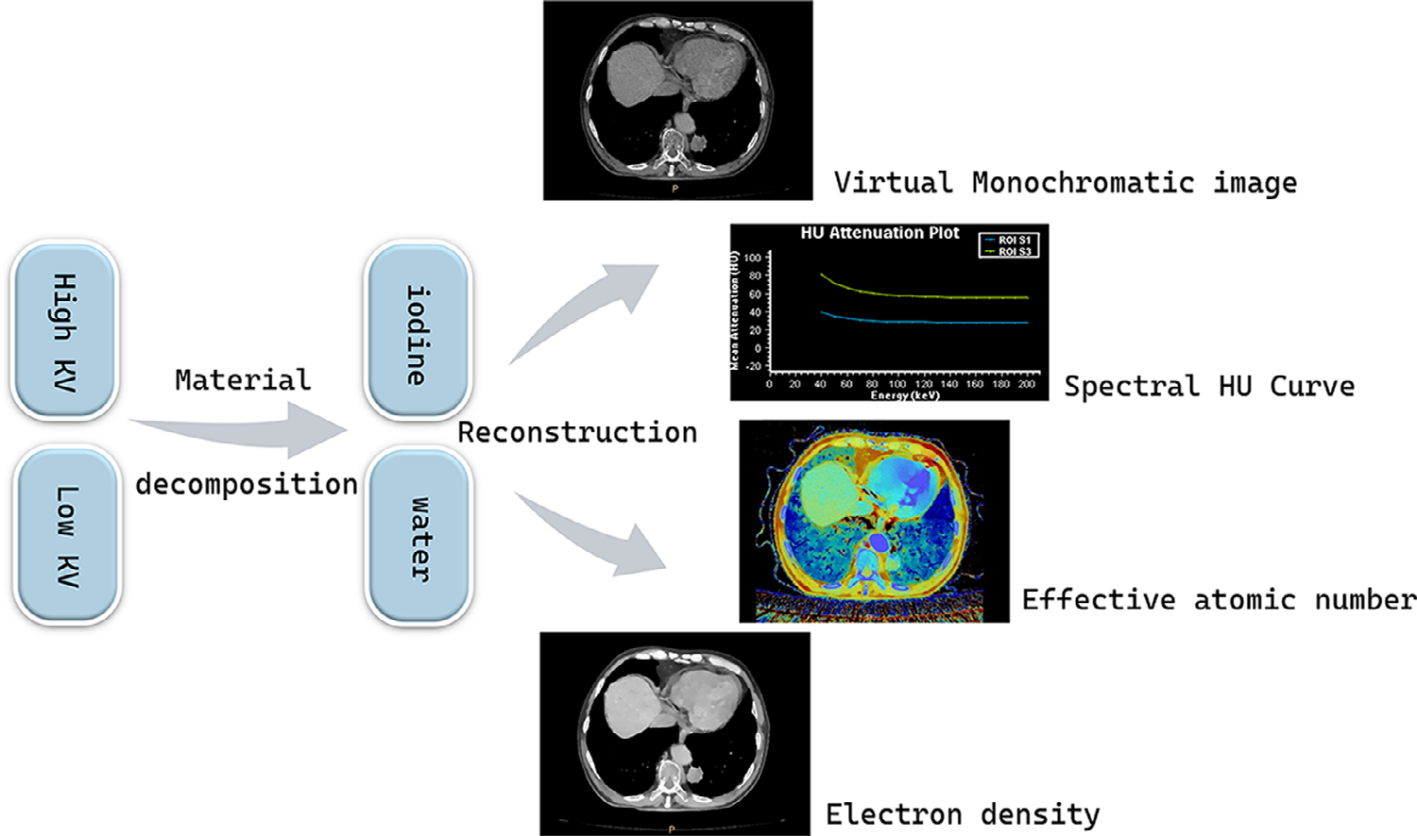
Raw data‐based approach for dual‐energy CT analysis.

VMI simulates CT images obtained with monochromatic x‐rays of any energy by decomposing two basic materials (usually iodine and water), and can generate up to 161 sets of monoenergetic images of 40–200  keV (Figure 4). In dual‐energy processing, the linear attenuation coefficient (μ) within a specific voxel can be expressed by the following formula:

(4) $$\mu \;\left(E_{m}\right)=\left(\rho_{p}\right)f_{p}\left(E_{m}\right)+\left(\rho_{c}\;\right)f_{c}\left(E_{m}\right)$$

Among them, $f_{p}\left(E_{m}\right)$ and $f_{c}\left(E_{m}\right)$ are functions that depend on the photoelectric effect and the Compton effect, and the mass densities ($\rho_{p},\rho_{c}$) of the two basic materials are estimated by material decomposition. The CT number at a certain energy level (keV) is defined by the following formula:

(5) $$CTnumber\;\left(E\right)=\frac{1000\left[\mu \left(E\right)-\mu_{water}\left(E\right)\right]}{\mu_{water}\left(E\right)}\;$$

where $\mu_{water}\left(E\right)$ is the linear attenuation coefficient of water. Based on these two formulas, CT numbers at any energy level (keV) can be obtained to reconstruct a virtual single‐energy image.

Low‐energy images (40–70 keV) enhance contrast and help detect small lesions; high‐energy images (80–200 keV) can reduce metal artifacts, although they will reduce the image contrast‐to‐noise ratio.^11^ Studies have shown that single‐energy images of 60 and 70 keV can provide high‐quality lung cancer imaging.^12^, ^13^

### 2.3 Energy spectrum curve

Define the region of interest (ROI) and measure the average CT value within this ROI at each discrete energy level to generate the corresponding energy spectrum curve. Different curve shapes reflect differences in tissue composition. Fat, iodine, and bone show significant differences in the energy spectrum curve, showing the clinical application value of lung cancer diagnosis and identification.^14^, ^15^, ^16^

### 2.4 Zeff and ED

Accurate acquisition of effective atomic number and electron density is critical for tissue identification. Zeff diagrams and ED diagrams obtained through matrix material decomposition technology add material composition information to each pixel, providing complementary information for the differential diagnosis of lung cancer and metastases.^17^ Electron density map can supplement the deficiencies of conventional CT and PET/CT in diagnosing NSCLC mediastinal lymph node metastasis.^18^

## 3 APPLICATION OF DECT COMBINED WITH RADIOMICS IN THE DIAGNOSIS OF LUNG CANCER

### 3.1 Predict benign and malignant pulmonary nodules

With the advancement of imaging technology, the detection rate of pulmonary nodules is increasing year by year. However, due to the similar characteristics of benign and malignant nodules on images (lobulated or spiculated edges), conventional CT imaging is often difficult to accurately distinguish. DECT combined with radiomics can deeply explore the relationship between imaging features and pathological changes through non‐invasive analysis, significantly enhancing the ability to distinguish benign and malignant pulmonary nodules. For example, the model established by Xu et al.^19^ based on radiomics features extracted from iodine‐based maps showed high diagnostic performance in distinguishing inflammatory and malignant pulmonary nodules, which is consistent with the experimental results of Xu et al.^20^ The joint model established by the latter shows high differential diagnosis ability. However, both of them only place ROI based on the maximum level rather than full volume analysis, which has certain deviations. The radiomics model based on DECT single‐level images developed by Liang et al. effectively differentiated benign and malignant pulmonary nodules, and the model combining arterial phase and venous phase imaging (AUC = 0.8772) significantly improved the diagnostic performance.^21^ The study by Xu et al. demonstrated that a model based on the optimal combination of radiomics scores derived from 65 keV arterial phase (AP) and venous phase (VP) images exhibited the best performance in distinguishing between benign and malignant solitary solid pulmonary nodules.^22^ Chang et al.’s study also emphasized the value of DECT in identifying benign and malignant pure ground glass nodules (pGGNs), with EDM and vacuole sign as independent risk factors, which provides a good basis for the early diagnosis of pulmonary nodules, providing important information for treatment decisions.^23^

### 3.2 Distinguish pathological types of lung cancer

Since different pathological subtypes have different representations and biological properties, accurately distinguishing the pathological subtypes of lung cancer at an early stage is beneficial to the adoption of precise clinical treatment plans and is of great significance to the prognosis of patients. Previous studies have confirmed the advantages of conventional CT parameters assisted radiomics in predicting histological subtypes and metastatic disease of primary malignant lung tumors.^24^ DECT multiparameter imaging provides more characteristic information to radiomics, thereby further improving the ability to identify pathological types of lung cancer. The results of Azour et al. suggest that radiomics signature analysis combined with DECT can provide discriminatory power beyond traditional iodine quantification analysis, especially in differentiating primary and metastatic lung tumors.^25^ Chen et al. retrospectively studied 129 patients with pathologically confirmed NSCLC to develop a clinical predictive model versus a radiomics model using normalized iodine concentration (NIC) and VMI, respectively, to discriminate lung adenocarcinoma (LUAD) from squamous cell carcinoma. The results showed that the clinical‐venous phase radiomics model had the best predictive performance (AUC of 0.93 and 0.90 for the training and validation cohorts, respectively), goodness of fit, parsimony, and clinical practicability for adenocarcinoma in NSCLC. It provides a relatively accurate, convenient, and non‐invasive method for predicting the pathological subtypes of adenocarcinoma and squamous cell carcinoma in NSCLC.^4^ Li et al. verified the feasibility of combining virtual plain scan images of DECT with radiomics to identify LUAD and squamous cell carcinoma (AUC of the training group and test group were 0.77 and 0.74, respectively),^26^ but this study did not make full use of the iodine concentration (IC) and energy spectrum curve slope obtained by post‐processing, which has certain limitations.

### 3.3 Predicting invasiveness of lung adenocarcinoma

Lung adenocarcinoma is a major type of lung cancer. Its early manifestations are ground glass nodules (GGN) on conventional CT. This feature often indicates LUAD or its precursors. Since the degree of invasion directly affects surgical strategies, accurate assessment of the invasiveness of LUAD is crucial for clinical guidance. Zheng et al. extracted radiomics features from virtual monoenergetic images (50 and 150  KeV) of 92 LUAD patients and found that the radiomics model was better than the traditional clinical model in predicting LUAD invasiveness (the training set and the test set AUCs are 0.957 vs. 0.929, 0.865 vs. 0.719, respectively).^1^ Chang et al. built a model based on multi‐modal radiomics of DECT, which effectively predicted the invasiveness of GGN.^27^ Zheng et al. demonstrated exceptional performance in predicting the pathological grade of invasive LUAD by developing a VMI‐based radiomics model, thereby underscoring the significant role of radiomics in the diagnosis and prognosis assessment of LUAD.^28^

### 3.4 Predicting biomarkers

Ki‐67 is a nuclear protein that is widely used in the diagnosis of lung cancer, breast cancer, and prostate cancer as a biomarker of tumor aggressiveness. It can not only distinguish the pathological types of lung cancer, but also predict the tumor growth rate through correlation with blood vessel volume.^29^ Studies have shown that multiple parameters of DECT are related to the Ki‐67 index, and three‐dimensional imaging features can comprehensively evaluate tumors and make up for the short‐comings of DECT in the assessment of tumor heterogeneity.^30^ In addition, PD‐1 and its ligand PD‐L1 cause tumor immune escape by inhibiting the activity of T cells. PD‐L1 protein expression is used to predict patient response to immunotherapy. Parameters of DECT have been used to differentiate PD‐L1 expression status in LUAD.^31^ Zheng et al. established a radiomics model to predict PD‐L1 expression levels by extracting radiomics features from DECT images and using logistic regression and support vector machine algorithms, demonstrating its application potential in NSCLC.^32^

### 3.5 Predicting lung cancer mutation status

Targeted therapy is an important method in the treatment of lung cancer, especially in the detection of common NSCLC mutations such as epidermal growth factor receptor (EGFR), rat sarcoma viral oncogene (KRAS), anaplastic lymphoma kinase (ALK), and BRAF genes.^33^ Previous studies have shown that quantitative parameters of DECT can effectively predict EGFR expression, which is better than conventional CT.^34^ Ma et al. retrospectively analyzed 173 patients with LUAD and established seven prediction models after extracting CT radiomics, DECT, and clinical features, and found that DECT combined with radiomics demonstrated significant superiority in predicting the EGFR mutation status of LUAD as compared with the prediction of DECT model alone through comparative analysis. In addition, this study provides an image‐based biological information‐assisted decision‐making tool for targeted therapy of EGFR‐mutant LUAD patients by integrating the nomograms constructed by the best models of clinical‐DECT‐radiomics.^35^ The DECT radiomics model developed by Wu et al. can not only non‐invasively predict the expression of VEGF and EGFR, but also reflect tumor angiogenesis and cell proliferation.^36^ Zhou et al. found that the EGFR mutation status of LUAD is related to the slope of the DECT spectrum curve, and the radiomics model constructed has good predictive performance.^37^ At present, there are no studies that combine radiomics to predict the mutation effects of ALK and BRAF genes, which will shed some light on future research.

### 3.6 Evaluate the treatment effect and prognosis of lung cancer

Current clinical treatment options for advanced lung cancer mainly include radiotherapy, chemotherapy, and targeted therapy, with chemotherapy being the most commonly used method. The internationally recognized response evaluation criteria in solid tumors (RECIST) have certain lags and limitations because it is only based on changes in tumor anatomy and morphology. Multiple studies have confirmed that IOM based on DECT can reflect tumor perfusion conditions to achieve early evaluation of chemotherapy efficacy.^38^, ^39^ Tanaka et al. found that tumors with lower IC are associated with higher recurrence rates after SBRT, showing that the application of IC in predicting radiotherapy effects has important implications for clinical decision‐making.^40^ Radiomics has great potential in assessing the prognosis of lung cancer patients by deeply mining image information. Choe et al. retrospectively studied 97 patients who underwent lung resection and extracted radiomic features through DECT iodine coverage. Using Cox proportional hazards regression models, they assessed the association of these characteristics with patient overall survival (OS) and disease‐free survival (DFS). The results showed that in multivariable analysis, regrouping by stage and radiomics parameters such as histogram entropy are independent risk factors for predicting DFS and OS, and the combination of the two provides higher prediction accuracy (C‐index: 0.72 for OS and 0.70 for DFS).^41^ In addition, Wu et al. developed a clinical‐radiomics nomogram utilizing the difference of ED and the variation in energy spectrum slope during the arteriovenous phase of pre‐treatment DECT images of advanced NSCLC. This model enables the personalized prediction of short‐term therapeutic responses to non‐surgical interventions.^42^ These findings emphasize the important value of DECT based on radiomics in the prognostic assessment of lung cancer patients, echoing the current pursuit of personalized treatment strategies in the field of oncology.

## 4 DECT IMAGING BASED ON DEEP LEARNING

Radiomics models are divided into traditional radiomics based on machine learning and radiomics based on deep learning. Traditional radiomics covers steps such as image acquisition, segmentation, feature extraction and quantification, statistical analysis, and model construction. The deep learning method uses an end‐to‐end neural network to learn directly from the original image without accurately segmenting the area of interest, effectively reducing human differences and costs, and has become the focus of research.^43^ For example, Wang et al. used a pre‐trained convolutional neural network to automatically segment lung nodules, and combined with radiomics features to improve the model's performance in predicting the invasiveness of LUAD.^44^ The model established by Yu et al. using artificial neural network (ANN) also confirmed that radiomics features have high diagnostic value in distinguishing lung cancer nodules from inflammatory nodules.^45^ The research of Vinay Kumar et al. also showed that the optimized U‐Net model significantly improved the speed and accuracy of automatic detection of lung cancer nodules, and solved the problem of feature instability caused by manually delineating ROI areas in traditional radiomics.^46^ Ma et al. found that AI algorithms trained on conventional CT showed consistent diagnostic performance on VMI, and 80 keV may be the best virtual monochromatic energy for identifying preoperative invasive adenocarcinoma on non‐enhanced chest CT.^47^ In addition, Wang et al. used Inception‐Res‐Net neural network and DECT VMI to build a prediction model, and used multi‐energy level fusion to improve the accuracy of predicting lung cancer lymph node metastasis to 93%, with a Kappa value of 86%.^48^, ^49^ Despite this, there are still few studies on combining deep learning models with DECT for lung cancer diagnosis, and further in‐depth exploration is needed.

## 5 APPLICATION OF PCCT IN LUNG CANCER

**FIGURE 5:**
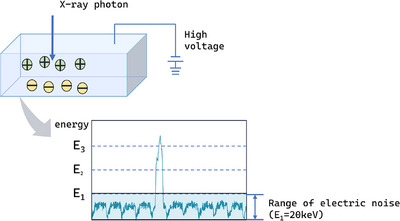
Incident x‐ray photons into the detector and generation of pulse (ideal situation). Electrical noise is eliminated by an energy threshold well above the electrical noise energy.

In recent years, photon‐counting CT (PCCT) has developed rapidly. Conventional energy spectrum CT mostly uses energy‐integrating detectors (EID). X‐rays are first converted into visible light, and then absorbed by the photodiode and generate charges, thereby generating image signals. In the photon counting detector (PCD), x‐ray photons are converted into electron‐hole pairs in semiconductor materials such as CdTe or CZT, and enter the anode electrode under the action of the electric field between the detectors to generate pulses. PCCT reads data in different energy domains by setting multiple thresholds (T0, T1, T2, T3) that are much higher than electronic noise (T0 threshold is usually about 20–25 keV), thereby improving the spatial resolution of CT images, reducing radiation dose and image noise.^50^, ^51^ (Figure 5) PCD technology has broad prospects in chest imaging. Studies have shown that PCCT scans provide better image quality and use significantly less radiation dose than EID‐CT scans, demonstrating advantages in the detection of lung nodules, classification of interstitial lung disease, and promise for low‐dose lung cancer screening.^52^, ^53^, ^54^ Although PCCT significantly affects the texture features of CT images by improving spatial resolution, thereby affecting the extraction of radiomics features,^55^ there is currently a lack of literature that combines PCCT with radiomics for lung cancer research. This gap provides new perspectives and potential directions for future radiomics research in lung cancer diagnosis and treatment.

## 6 CONCLUSION

As an emerging imaging technology, DECT can provide quantitative measurement of multiple parameters. It has obvious advantages in the detection and identification of lung cancer and the prediction of biomarkers and gene mutations, making up for the shortcomings of conventional CT. Radiomics processes large amounts of imaging information in an automated, high‐throughput manner. Its combination with DECT shows broad application prospects in the field of lung cancer. However, current research still has the following problems: (1) Single‐center retrospective studies and large sample sizes lead to bias. Small sample sizes (most current research sample sizes are around 100) may lead to representative and generalized research results. Limited imaging capabilities (2) Traditional radiomics mostly uses manual marking of ROIs. Due to differences in personal clinical experience, it inevitably affects the extraction of radiomics features. (3) Problems such as incomplete research coverage. Currently, there is a lack of research on the effect of combining radiomics to predict ALK and BRAF gene mutations. There are still few relevant studies on predicting PD‐L1 expression status in LUAD. There is a gap in the application of PCCT combined with radiomics in the diagnosis and identification of lung cancer. Nonetheless, I believe that with the continuous deepening of clinical applications and the development of artificial intelligence, the acquisition of large samples, standardized data, and the application of automated precise segmentation algorithms will become possible. DECT combined with radiomics is expected to exert unique advantages in multiple fields of tumor treatment, supporting precision medicine with low cost, low radiation, high efficiency, and high accuracy.

## AUTHOR CONTRIBUTIONS

**Rongyu Zhang**: Conceptualization; writing—original draft; preparation. **Hao Zheng**: Conceptualization; writing—review and editing. **Jie Lin**: writing—review and editing. **Junna Wang**: writing—review and editing; supervision. All authors read and approved the final manuscript.

## CONFLICT OF INTEREST STATEMENT

The authors declare no conflicts of interest.
